# Efficacy of bi-level erector spinae plane block versus bi-level thoracic paravertebral block for postoperative analgesia in modified radical mastectomy: a prospective randomized comparative study

**DOI:** 10.1186/s12871-023-02157-2

**Published:** 2023-06-16

**Authors:** Domenico P. Santonastaso, Annabella de Chiara, Roberto Righetti, Diego Marandola, Andrea Sica, Claude T. Bagaphou, Chiara Rosato, Andrea Tognù, Annalisa Curcio, Leonardo Lucchi, Emanuele Russo, Vanni Agnoletti

**Affiliations:** 1 Anesthesia and Intensive Care Unit, AUSL Romagna, M.Bufalini Hospital, Viale Ghirotti 286-47521, Cesena, Italy; 2 Anesthesia and Intensive Care Unit, AUSL Romagna, Santa Maria Delle Croci Hospital, Viale Randi 5, 48121 Ravenna, Italy; 3 Section of Anesthesia, Intensive Care and Pain Medicine, Ospedale Di Città Di Castello - USL Umbria1, Città Di Castello, Perugia, Italy; 4 Section of Anesthesia and Intensive Care Unit, Istituto Ortopedico Rizzoli, Ospedale Mazzolani Vandini, Via Nazionale Ponente, 7, 44011 Argenta, Italy; 5 General Surgery Unit, AUSL Romagna, Santa Maria Delle Croci Hospital, Viale Randi 5, 48121 Ravenna, Italy; 6 Day Surgery – Breast Unit, AUSL Romagna, M.Bufalini Hospital, Viale Ghirotti, 286-47521 Cesena, FC Italy

**Keywords:** Thoracic paravertebral block, Erector spinae plane block, Breast surgery, Mastectomy, Postoperative pain

## Abstract

**Background:**

Postoperative analgesia in breast surgery is difficult due to the extensive nature of the surgery and the complex innervation of the breast; general anesthesia can be associated with regional anesthesia techniques to control intra- and post-postoperative pain.

This randomized comparative study aimed to compare the efficacy of the erector spinae plane block and the thoracic paravertebral block in radical mastectomy procedures with or without axillary emptying.

**Methods:**

This prospective randomized comparative study included 82 adult females who were randomly divided into two groups using a computer-generated random number. Both groups, Thoracic Paraverterbal block group and Erector Spinae Plane Block group (41 patients each), received general anesthesia associated with a multilevel single-shot thoracic paravertebral block and a multilevel single-shot erector spinae plane block, respectively.

Postoperative pain intensity (expressed as Numeric Rating Scale), patients who needed rescue analgesic, intra- and post-operative opioid consumption, post-operative nausea and vomiting, length of stay, adverse events, chronic pain at 6 months, and the patient’s satisfaction were recorded.

**Results:**

At 2 h (*p* < 0.001) and 6 h (*p* = 0.012) the Numeric Rating Scale was significantly lower in Thoracic Paraverterbal block group.

The Numeric Rating Scale at 12, 24, and 36 postoperative hours did not show significant differences.

There were no significant differences also in the number of patients requiring rescue doses of NSAIDs, in intra- and post-operative opioid consumption, in post-operative nausea and vomiting episodes and in the length of stay.

No failures or complications occurred in the execution of techniques and none of the patients reported any chronic pain at six months from the surgery.

**Conclusions:**

Both thoracic paravertebral block and erector spinae plane block can be effectively used in controlling post-mastectomy pain with no significant differences between the two blocks.

**Trial registration:**

The study was prospectively registered on Clinicaltrials.gov (trial identifier NCT04457115) (first registration 27/04/2020).

## Background

Breast cancer is the most common cancer among women [1] and breast surgery is often required to remove the primary tumor as well as axillary staging or dissection.

Approximately half of women who undergo minor or major breast surgery describe an important postoperative pain (> 5 on the Visual Analogue Scale, VAS) which is not always effectively controlled by standard post-operative therapies [2].

Post-operative analgesia in breast surgery is difficult due to the extensive nature of the surgery and the complex innervation of the breast and poor postoperative pain control may produce a series of acute and chronic complications [3, 4].

Associating general anesthesia (GA) with regional anesthesia techniques, improves pain control, reduce the perioperative need of analgesic drugs, in particular reducing opioid requirements during the perioperative period according with the enhanced recovery programs, diminish postoperative nausea/vomiting (PONV), help in reducing the development of chronic pain, and facilitate early rehabilitation [5].

Although using regional blocks as an adjunct to analgesia existed for many years, they have only recently increased in popularity as a method of postoperative pain management.

Pectoralis blocks (PECS 1 and PECS 2), Serratus anterior plane (SAP) block [6], thoracic paravertebral (TPV) block [7] and Erector spinae plane (ESP) block [8] have been successfully used for perioperative analgesia following breast surgeries.

Chronic pain is a problem that afflicts about half of women undergoing radical mastectomy [9] and the use of regional anesthesia allows a reduction in the onset and severity of chronic pain [10].

Proposed mechanisms for decreasing persistent pain include decreasing central sensitization (wind-up) and reducing opioid-induced hyperalgesia [11].

The role of local anesthetics (LA) given for the peripheral nerve block in affecting post-operative nerve impulse activity, in slowing the changes in synaptic neuroplasticity, or in changing the signaling properties of non-neuronal cells has been debated for the past two decades [12, 13].

Furthermore, regional anesthesia may reduce cancer progression by attenuation of the surgical stress response, better analgesia, and reduced opioid usage, and by the direct protective action of local anesthetics on migration of cancer cells [14].

This randomized comparative study, which was carried out on 82 patients who underwent radical mastectomy, aims to compare the efficacy of TPV block with a simpler technique to perform such as the ESP block.

Primary aim was comparing the efficacy of TPV block and ESP block on the postoperative pain intensity expressed as Numeric Rate Scale (NRS).

## Methods

This prospective, randomized comparative study was conducted at M. Bufalini Hospital, Cesena, Italy, and Santa Maria delle Grazie Hospital, Ravenna, Italy, from April 2020 to November 2021.

It included 82 adult females, aged 18 to 90 years with an American Society of Anesthesiologists (ASA) risk ranging from I to IV, with no contraindications for the execution of TPV and ESP blocks, scheduled for elective modified radical mastectomy (MRM) with or without axillary dissection.

The study was approved by the ethics committee of the AUSL (Azienda Unità Sanitaria Locale- local health authority) of Romagna (Ref. 2149/2020 I.5/299) and IRST (Istituto Romagnolo Studio Tumori—Institute for Cancer Study of Romagna) and was prospectively registered on Clinicaltrials.gov (trial identifier NCT04457115, first registration 27/04/2020).

The full trial protocol is available from the corresponding author.

Patients with allergies and/or contraindications for the administration of drugs used in the study were excluded from the study, as well as patients who presented with chronic opioid use for therapeutic purposes, patients with coagulopathies and/or who used antiaggregant or anticoagulant drugs, with infections and lesions at the puncture site or with a Body Mass Index (BMI) ≥ 40.

The primary outcome for this study was NRS pain score 12 h after surgery.

The secondary outcomes were:

- NRS pain score at different time point: at the awakening of the patients (time 0) and 2, 6, 24 and 36 post-operative hours
- Patients who needed rescue analgesic (non-steroidal anti-inflammatory drugs—NSAIDs)
- Intra- and post-operative opioid consumption
- PONV
- Length of stay
- Patients with adverse events (accidental vascular puncture, accidental pneumothorax, nerve damage, and Horner’s/Harlequin’s syndrome)
- Post-operative chronic pain assessment (6 months)
- Patients’ satisfaction regarding anesthesiologic procedure

All patients were admitted to the hospital after having followed an anesthetic visit in pre-hospitalization in the morning on the day of surgery and received premedication with midazolam 3 mg intramuscularly one hour before the surgery, only if requested by the patient.

Furthermore during the visit, the patients were instructed on what is meant by NRS scales.

In all cases the same six anesthesiologists performed the blocks and the same two surgeons performed the surgery.

In the pre-anesthesia holding area, standard monitoring, including non-invasive blood pressure, electrocardiogram, pulse oximetry, and Bispectral Index System (BIS, Covidien Medtronic, Minneapolis, MN, USA) were applied.

Both blocks was performed thirty minutes before the surgery with a sterile technique and out of plane approach using hydrolocation with 0.9% saline to help show the position of the needle tip, with the patients placed in the lateral position with the surgical site being uppermost.

The ultrasound guidance (SonoSite M-Turbo, SonoSite Inc., Bothell, WA), with a 16–6 MHz linear probe and 22-G × 50-mm needle (Echoplex + , Vygon, Ecouen-France) positioned into a longitudinal orientation to obtain a para-sagittal view, was used for both the blocks.

### TPV block

**Fig. 1:**
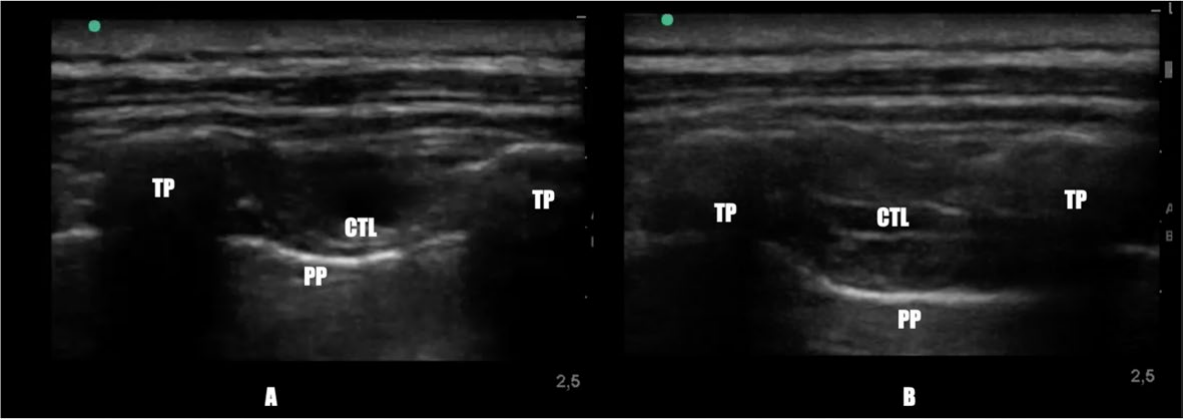
Thoracic paravertebral space, before (**A**) and after (**B**) execution of thoracic paravertebral block. TP: Transverse Process; PP: Parietal Pleura; CTL: Superior Costo-transverse ligament

TPV Block was performed on two thoracic (T) levels (T2- T3 and T4-T5); ultrasound guidance was used to visualize the superior costo-transverse ligament and the pleura as hyperechoic structures, and the paravertebral space was visualized as a wedge-shaped hypoechoic layer between these structures (Fig. 1).

After local infiltration with 2.0 mL of 2% lignocaine at the site of puncture, the block was performed at two thoracic levels, and for each level 8 mL of 0.75% ropivacaine were injected (120 mg of Ropivacaine), not exceeding the toxic dose.

The spread of local anesthetic in the paravertebral space and a concomitant anterior movement of the parietal pleura were observed using real-time image guidance.

### ESP block

Ultrasound guided ESP Block was performed, after local infiltration with 2.0 mL of 2% lignocaine at the site of puncture, on two thoracic (T) levels (T2 and T5) using the transverse process, visualized as a hyperechoic structure with acoustic shadowing below, as main ultrasound landmark.

**Fig. 2:**
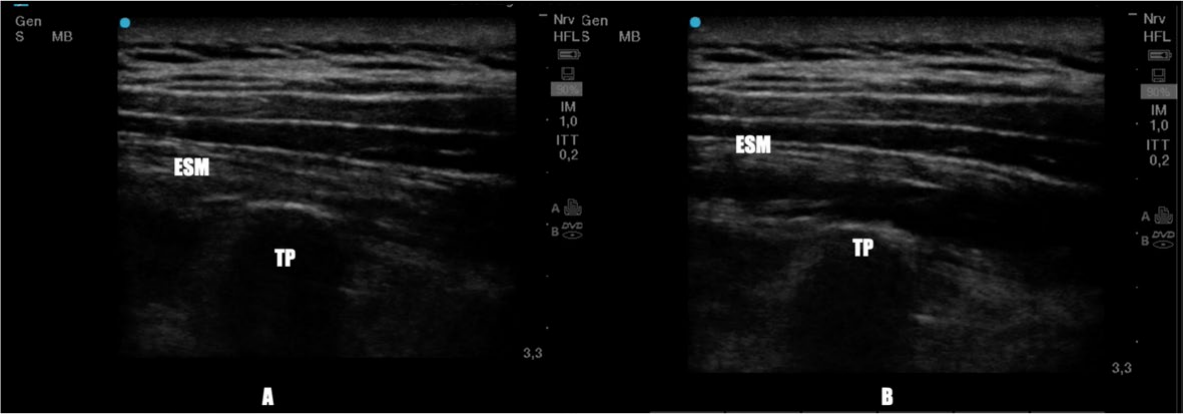
Erector spinae muscle before (**A**) local anesthetic administration and after (**B**) local anesthetic administration. TP: Transverse process; ESM: Erector Spinae Muscle

When the needle came in contact with the transverse process 12 mL of 0.5% ropivacaine was injected for each level (120 mg of Ropivacaine), not exceeding the toxic dose (Fig. 2).

Thirty minutes after performing the blocks, the sensory level of the block was assessed by a cold sensation with an alcohol–soaked sponge and a pin prick testing using a 22-G short bevel needle, in each dermatomal distribution from T1 to T8 by an anesthesiologist who was not aware of the study group.

All patients had dermatomal coverage from T1 to T8.

All patients in Thoracic Paraverterbal block group (Group TPVB) and Erector Spinae Plane Block group (Group ESPB) received GA with intravenous (IV) bolus of propofol 2 mg kg^−1^ and rocuronium 0.6 mg kg^−1^, and I-Gel (Intersurgical, Berkshire, UK) was inserted according to the manufacturer’s instruction.

IV dexamethasone 8 mg was administered before induction of anesthesia.

Anesthesia was maintained by continuous IV infusion of propofol (6–9 mg kg^−1^ h^−1^), and the sedation level was monitored using BIS.

Fentanyl 100 mcg in bolus was administered intravenously if the mean blood pressure or heart rate exceeded 20% of the pre-operative value. The total intraoperative fentanyl consumption was recorded.

In patients requiring an axillary lymph node dissection, top-up local infiltration analgesia into the surgical field, to allow the enlargement of the operative field, was performed by the surgeon using 6 mL lignocaine (20 mg mL^−1^).

Hypotension (mean arterial pressure < 60 mmHg) was treated with boluses of fluid and, if required, ephedrine in doses of 25–50 mg. Bradycardia (HR < 40 beats min or < 20% of baseline) was managed with 0.5 mg atropine.

All patients were administered 2 gr of Cefazolin half an hour before the start of surgery.

No prophylactic antiemetics were administered.

Acetaminophen 1gr IV was administered 30 min before the end of surgery, and then every eight hours.

The pain assessment, at rest and related to movement, was carried out, using the NRS scale, at the awakening of the patients (time 0) and 2, 6, 12, 24 and 36 post-operative hours. The assessment was done by an anesthesiologist who had no role in giving the block and in the intraoperative management of the patient but aware of the type of block performed.

Wherever pain exceeded 3 according to the NRS scale, or at the explicit request of the patient, 30 mg of Ketorolac was administered IV for a maximum of three times a day.

We used morphine 2 mg if, thirty minutes after the administration of Ketorolac, pain persists with NRS > 3.

A total of 492 NRS and dose rescue request measurements were performed.

The first-line treatment of PONV consisted of IV ondansetron 4 mg twice a day as needed and, if this proved ineffective, second-line therapy consisted of IV metoclopramide 10 mg as needed.

The quantity of opioids (morphine) administered post-operatively, requests for rescue doses of NSAIDs, and the presence and number of nausea and vomiting episodes, were all assessed.

Patients’ satisfaction was evaluated and recorded 36 h after surgery on a 7‐point Likert scale. (1‐ Extremely dissatisfied, 2‐ Very dissatisfied, 3‐ Dissatisfied, 4‐ Neither satisfied nor dissatisfied, 5‐ Satisfied, 6‐ Very satisfied, 7‐ Extremely satisfied).

The patients were then discharged when deemed safe according to the Italian version of the Post Anesthesia Discharge Scoring System (PADS). Lastly, the total number of days of hospitalization were also recorded.

After six months, patients received a telephone call to ascertain the presence of chronic pain during rest and in motion assessed using the NRS. Patients were also asked to evaluate the pain interference with sleep, work activity, mood tone, and possibility of entertainment.

The sample size of the study was calculated based on a retrospective pilot study designed to measure NRS pain score 12 h after surgery in the two groups TPVB vs ESPB (10 patients in each group). We observed a mean NRS of 2.7 vs.0.9 (s.d. = 2.9). Setting the power to 80% and the alpha to 0.05%, the sample size required was 82 patients.

### Statistical analysis

Data were reported as mean with standard deviation (s.d.), median with interquartile range (IQR), and number and percentage depending on the underlying distribution.

Normality was test with Shapiro–Wilk test.

Differences between the two groups were tested with the t-test or Mann–Whitney according to the characteristics and distribution of the variables; for categorical ones, the Fisher’s exact test was used.

## Results

**Fig. 3:**
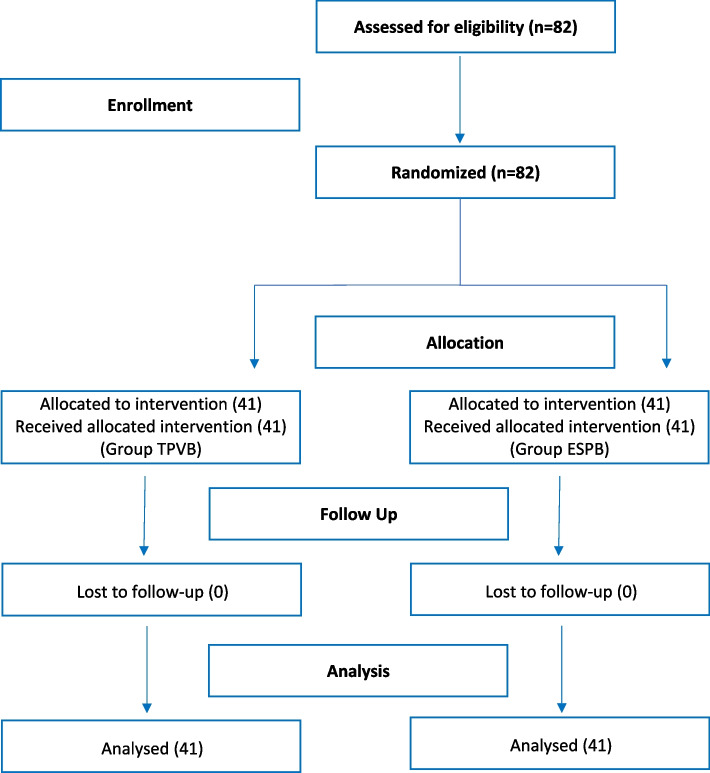
Consort Flow diagram

Eighty-two patients, randomized into two groups using a computer-generated random number (simple randomization method), were included in the study (41 per group), from June 2020 to November 2021, and no patient was excluded for any reason (Fig. 3).

Both the groups received GA, the group TPVB (41 patients) was associated with a single-shot TPV block and the Group ESPB (41 patients) was associated with a single-shot ESP block.

There were no significant differences between the two groups in age, sex, BMI, and ASA risk (Table 1).

**Table 1:** Demographic profile of patients and type of surgery

**PARAMETERS**		**Group TPVB (41)**	**Group ESPB (41)**	***p* Value**
AGE	Mean (sd)	68.1 (± 14.6)	68 (± 11.1)	0.981
	Median (IQR)	72 (23.5)	69 (17.5)	
BMI	Mean (sd)	24.5 (± 5.3)	25.9 (± 6.4)	0.283
	Median (IQR)	23.4 (4.1)	24.2 (5.1)	
ASA Physical Status	N (%)			0.191
ASA 1		8 (19.5)	3 (7.32)	
ASA 2		18 (43,9)	25 (61.0)	
ASA 3		15 (36.6)	12 (29.3)	
ASA 4		0 (0)	1 (2.44)	
Type of surgery	N (%)			0.413
M		14 (34.1)	19 (46.3)	
M + ALND		4 (9.76)	5 (12.2)	
M + SLND		23 (56.1)	17 (41.5)	

Age and BMI were tested by independent T student test

ASA and Type of Surgery were tested by Chi-Square test

*M* mastectomy, *SLND* Sentinel lymph-node dissection, *ALND* axillary lymph node dissection

Thirty-three patients underwent only mastectomy, fourteen in Group TPVB (34.1%) and nineteen in Group ESPB (46.3%) (Table 1).

Forty patients also underwent sentinel node biopsy, twenty-three in Group TPVB (56.1%) and seventeen in Group ESPB (41.5%); nine patients also underwent axillary dissection, four in Group TPVB (9.76%) and five in Group ESPB (15.2%) (Table 1).

There were no cases of sympathetic block.

Surgery was completed within a time range of 70 to 110 min.

Two patients in Group ESPB (4.88%) and 0 in Group TPVB presented with hypotension (*p* = 0.152). (Table 2).

**Table 2:** Clinical outcomes of the studied groups

Variables	Group TPVB	Group ESPB	*P*-value
**NRS on awakening**			0.808
Median (IQR)	0.85 (1.47)	0 (2)	
**NRS at 2 h**			< 0.001
Median (IQR)	1.7 (1.5)	2 (2)	
**NRS at 6 h**			0.012
Median (IQR)	1.46 (1.3)	2 (2)	
**NRS at 12 h**			0.089
Median (IQR)	0.56 (0.8)	0 (1)	
**NRS at 24 h**			0.429
Median (IQR)	0.12 (0.3)	0 (0)	
**NRS at 36 h**			1.0
Median (IQR)	0 (0)	0 (0)	
**Rescue dose on awaking** [N (%)]	2 (4.88)	0 (0)	0.152
**Rescue dose at 2 h** [N (%)]	5 (12.2)	6 (14.6)	0.745
**Rescue dose at 6 h** [N (%)]	4 (9.76)	4 (9.76)	1.00
**Rescue dose at 12 h** [N (%)]	2 (4.88)	0 (0)	0.152
**Rescue dose at 24 h** [N (%)]	1 (2.44)	0 (0)	0.314
**Rescue dose at 36 h** [N (%)]	1 (2.44)	0 (0)	0.314
**Intraoperative Fentanyl** [N (%)]	13 (31.7)	19 (46.3)	0.174
**PONV** [N (%)]	2 (4.88)	0 (0)	0.157
**Hypotension** [N (%)]	0 (0)	2 (4.88)	0.152
**Chronic pain** [N (%)]	0 (0)	0 (0)	1.0
**Length of stays**			1.0
Mean (s.d.)	2.4 (± 0,5)	2.3 (± 0,6)	

Rescue dose, Intraoperative Fentanyl, PONV, Hypotension, Chronic pain and length of stay were tested by Chi Square test

NRS was tested by U di Mann–Whitney test

*S.d.* standard deviation, *IQR* interquartile range, *N* number of patients, *NRS* Numeric rating scale, *PONV* post operative nausea and vomiting

Thirty-two patients required intra-operative use of fentanyl (100 mcg), thirteen in Group TPVB (31.7%) and nineteen in Group ESPB (46.3%) (*p* = 0.152) (Table 2).

No post-operative opioid was used for any patient.

The differences in the rescue dose requirement did not reach significance in the two groups.

**Fig. 4:**
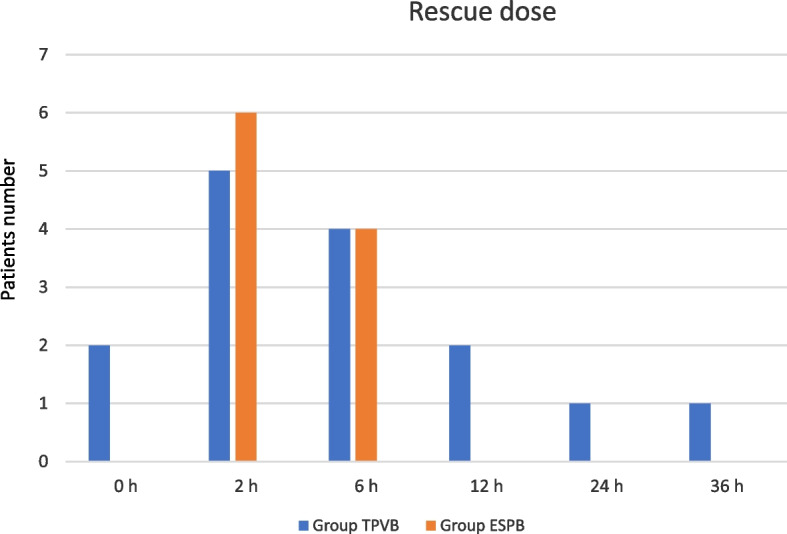
Rescue dose patients request at awakening (0 h) and at 2–6-12–24-36 post-operative hours

The major percentage of the rescue dose was administered at 2 h, five patients in Group TPVB (12.2%) and six in Group ESPB (14.6%), with no significant difference between the two groups (Table 2) (Fig. 4).

The NRS, assessed at immediate post-operative (0 min) and at 2, 6,12, 24 and 36 post-operative hours, was higher than 3 only sixteen times out of a total of 492 measurements (3.25%) (Table 2).

The mean and median of the NRS were always less than 3 for all measurement groups.

At 2 and 6 h NRS was significantly lower in Group TPVB.

At 2 h mean NRS was 0.78 (± 1.4) in Group TPVB and 1.7 (± 1.5) in Group ESPB, (*p* < 0.001).

**Fig. 5:**
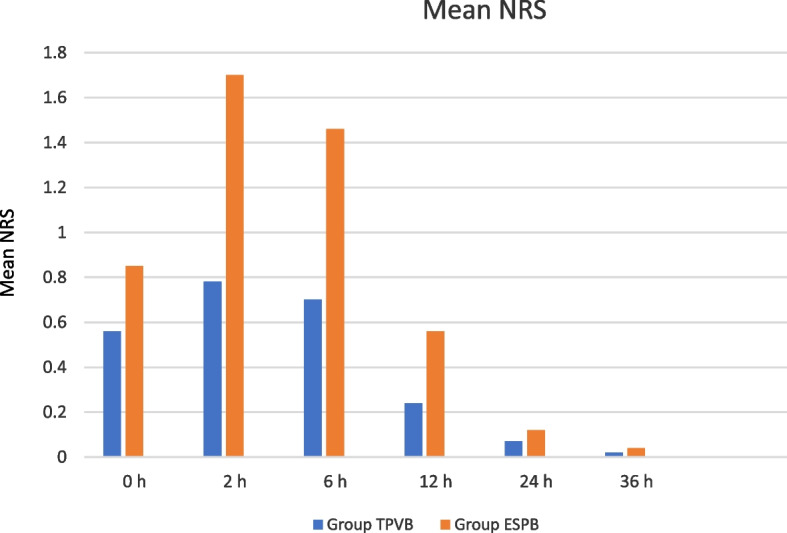
Mean NRS at awakening (0 h) and at 2–6-12–24-36 post-operative hours

At 6 h mean NRS was 0.70 (± 1.0) in Group TPVB and 1.46 (± 1.3) in Group ESPB, (*p* = 0.012) (Table 2) (Fig. 5).

Two episodes of PONV were observed in Group TPVB (4.88%), that resolved spontaneously without the use of antiemetic drugs, and no episode of PONV was seen in Group ESPB (*p* = 0.152) (Table 2).

There was no significant difference in the length of stay (Table 2).

All patients expressed a satisfaction level of 7 with the anesthesiologic procedure (very satisfied).

No failures or complications occurred in the execution of both techniques.

None of the patients reported any chronic pain at six months from the surgery.

## Discussion

In our study the primary end point was to compare the TPV and ESP blocks in the control of post-operative pain after radical mastectomy with or without axillary emptying.

As secondary end points, we evaluated the need of rescue analgesia, intra- and post-operative opioid consumption, PONV, length of stay, adverse events, chronic pain after 6 months and patients satisfaction regarding anesthesiologic procedure.

We performed both TPV and ESP blocks on two levels. We highlight the importance to “block” the T2 nerve root because from the T2 spinal level originates the inter-costobrachial nerve, which is very important for the axilla innervation.

Both the regional blocks reduced the intra- and post-operative opioid consumption, with a comparable duration of analgesic effect and stable hemodynamic profiles.

At 2 and 6 postoperative hours, the NRS was significantly lower in Group TPVB than in Group ESPB; however, this difference did not lead to an increased request for rescue doses by patients.

The NRS at 12, 24, and 36 postoperative hours did not show significant differences.

The number of patients requiring intra-operative opioid administration (100 mcg of Fentanyl) was higher among those in Group ESPB; however, we found no statistically significant differences.

No statistically significant data were recorded regarding the PONV episodes and length of stay.

We did not observe any adverse events (through a clinical assessment of the patients), which is probably related to the great experience of the operators. All patients were absolutely satisfied with the anesthetic procedure and none developed chronic pain 6 months after surgery.

Even if not included in our primary and secondary end points, we underline that there were no significant differences in the execution times of the two blocks.

Despite the large number of analgesic modalities for postoperative pain management among women undergoing breast surgery, recent reports still highlight a significant post-operative pain burden in this population [15].

Breast surgery is burdened by a high incidence of acute post-operative pain whose inadequate management increases the risk not only of chronic pain but also of in-hospital mortality and functional impairments [16].

The analgesic and anesthetic effect of the TPV block is due to the direct contact of the LA injected into the paravertebral space with the roots of the spinal nerves as well as the spread in the epidural space. Thus, a thoracic paravertebral injection of LA results in ipsilateral somatic and sympathetic nerve blocks including the posterior ramus in multiple contiguous thoracic dermatomes [17].

However, the risk of pneumothorax, the long time to perform the block, and the high level of skills needed make some inexperienced anesthesiologists to avoid the TPV block and use other techniques, such as the ESP block, a novel nerve-blocking technique first proposed by Forero et al. in 2016 [18].

It is generally implemented through deposition of drugs into the fascial plane beneath the erector spinae muscle at the tip of the transverse process of the vertebra, thereby reducing the risk of pneumothorax and significant neurovascular damage.

The ESP block allows anesthetic coverage similar to that of the TPV Block. It is considered a peri-paravertebral regional anesthesia technique which is supposed to block the dorsal and ventral rami of the thoracic and abdominal spinal nerves, and thereby block the anterior, posterior, and lateral thoracic and abdominal walls.

The mechanism of action of the ESP block is probably related to the spread of the LA into the paravertebral space [19].

This hypothesis is reinforced by the recent discovery that identifies two slits at the medial and lateral ends of the superior costo-transverse ligament (SCTL) that can act as channels to the thoracic paravertebral space [20].

The TPV block is certainly one of the most effective techniques in pain control after radical mastectomy [7, 21], and in the work of Jacobs et al. it is considered as the gold standard for major breast surgery [22].

Several previous studies, though, have documented that ultrasound-guided paravertebral block is an advanced regional anesthetic technique that requires a longer learning curve to manipulate the needle under ultrasonography guidance towards the paravertebral space [23].

Krediet et al. [24], in their review of the TPV space, concluded that at least nine approaches are available for the TPV block; we preferred the out of plane approach because of our personal preference.

Nevertheless, the complications of TPV block like inadvertent pleural puncture and epidural or intrathecal spread are still a concern even with ultrasound utilization.

Ultrasound-guided ESPB, which we preferably perform with an out of plane technique, has been originally described for pain relief in patients with chronic neuropathic pain. However, recent studies found it effective as a postoperative analgesic technique and to reduce the postoperative opioid consumption following breast surgery.

Zhang et al. [25] in their meta-analysis, which evaluates as main outcome opioid consumption within the first 24 h after surgery and as secondary objectives pain scores after surgery, intra-operative opioid consumption, the incidence of PONV and block-related adverse events, revealed that ultrasound-guided ESP block provided better post-operative pain control by reducing peri-operative opioid consumption and VAS pain scores in patients after breast cancer surgery, in comparison to GA alone.

Agarwal et al. [26] concluded their study highlighting that ESP and TPV blocks are comparable in terms of post-operative analgesia in MRM; however, ESP block can be used as a safer and alternative analgesic technique to perform over TPV block in breast cancer surgery.

Unlike our study, they did not find statistically significant differences in postoperative NRS scores at rest and movement at 2 and 6 post-operative hours.

The same observations were reported by Moustafa et al.[27] in their study: ESP and TPV blocks exhibited no significant differences in the opioid-sparing effects among women undergoing MRM. Xiong et al., in their meta-analysis, underlined how the post-operative analgesic effects of TPV and ESP blocks were similar [28].

Most of the studies comparing TPV and ESP blocks in breast surgery involved the use of post-operative morphine while none of the patients in our study used post-operative morphine.

The use of opioids, intra- and post-operative, in addition to having adverse effects such as nausea, vomiting, and constipation may potentiate the tumor cell survival and angiogenesis, which could lead to metastasis of cancer [29–31].

In our hospital the TPV block has been performed for many years for both mastectomies and quadrantectomies, therefore, its effectiveness has been extensively confirmed even for interventions with awake patients [7, 21, 32].

### Limitations of the study

The limitations of this study were the lack of a control group, the small number of cases performed and that we did not use patient controlled analgesia (PCA) pump, which could help standardize the administration of analgesics in the post-operative period.

## Conclusions

From the data obtained by our randomized study, we can confirm, as already underlined by the existing literature, that the TPV and ESP blocks used for radical mastectomy were absolutely effective, and very similar in the management of intra- and post-operative pain, in intra- and post-operative opioid consumption and length of stay.

It would be desirable to have a randomized trial comparing the TPV, ESP and PECS blocks, the most commonly used regional anesthesia techniques in major breast surgery.

## Data Availability

The data that support the findings of this study are available from the corresponding author upon reasonable request.

## Abbreviations

VAS: Visual Analogue Scale
GA: General anesthesia
PONV: Postoperative nausea/vomiting
PECS 1 and PECS 2: Pectoralis blocks
SAP: Serratus anterior plane
TPV: Thoracic paravertebral
ESP: Erector spinae plane
LA: Local anesthetics
NRS: Numeric Rate Scale
MRM: Modified radical mastectomy
BMI: Body Mass Index
NSAIDs: Non-steroidal anti-inflammatory drugs
BIS: Bispectral Index System
IV: Intravenous
PCA: Patient controlled analgesia
